# Identification of Key Pathways Involved in White Strain of *Hypsizygus marmoreus* Extracts-Induced Cell Death of Human Hepatoma Hep3B Cells by Next Generation Sequencing

**DOI:** 10.3389/fphar.2022.888863

**Published:** 2022-06-16

**Authors:** Wei-Sung Li, Kun-Tsung Denzel Lee, Li-Yun Chen, Bang-Jau You, Hong-Zin Lee

**Affiliations:** ^1^ Plant Pathology Division, Taiwan Agricultural Research Institute, Council of Agriculture, Executive Yuan, Taiwan; ^2^ Department of Chinese Pharmaceutical Sciences and Chinese Medicine Resources, China Medical University, Taichung, Taiwan; ^3^ Department of Oral Hygiene, College of Dental Medicine, Kaohsiung Medical University, Kaohsiung, Taiwan; ^4^ Department of Dentistry, Kaohsiung Medical University Hospital, Kaohsiung Medical University, Kaohsiung, Taiwan; ^5^ School of Pharmacy, China Medical University, Taichung, Taiwan

**Keywords:** white genius mushroom, human Hep3B liver cancer cells, transcriptome, autophagy, mitophagy, reactive oxygen species

## Abstract

White strain of *Hypsizygus marmoreus* is named as white genius mushroom (WGM) and is a popular food in Taiwan. We have confirmed the cytotoxicity of WGM extracts on human Hep3B liver cancer cells. A total of 8711 significantly differential genes were identified through large-scale transcriptome sequencing. According to the KEGG pathway enrichment analysis, autophagy, mitophagy and apoptosis pathways were identified as significant in WGM extracts-treated cells. WGM extracts induced a dose-dependent generation of reactive oxygen species (ROS) and membrane-enclosed vacuoles in Hep3B cells. The inhibition of ROS by the ROS scavengers blocked the induction of cell death and vacuoles formation. We suggested that the cell death and membrane-enclosed vacuoles induced by WGM extracts are dependent on ROS production in Hep3B cells. (2*E*,6*E*)-3,7,11,15,19,23,27,31,35-Nonamethylhexatriaconta-2,6,34-triene-1,11,15,19,23,27,31-heptol and (18:2) lysophosphatidylcholine were identified in WGM extracts. In addition to being a very popular edible mushrooms, WGM may be developed into a dietary supplement or dietary chemopreventive agent for the cancer treatment.

## Introduction

Recently, transcriptome profiling is a method of large-scale investigation of gene expression levels and has been widely used to identify cellular states, to find genes with similar expression patterns or to investigate molecular mechanisms of action of anticancer agents. The differentially expressed genes screened by RNA-sequencing can offer a new insight into the complex mechanisms of action of anticancer drugs (Wacker et al., 2012; Yang et al., 2013). RNA-sequencing is a technique for transcriptome profiling that uses next generation sequencing (NGS) technologies and measures transcript levels. The gene ontology (GO) term enrichment was applied to find the biological functions of differentially expressed genes, and the kyoto encyclopedia of genes and genomes (KEGG) pathway enrichment analysis was utilized to determine the related signal pathways. NGS is now generally accepted as a method to analyze total mRNA expression and elucidate cellular processes at the molecular level. Therefore, NGS allowed the identification of transcript levels that are involved in the induction of cell death in cancer cells to find the biological functions of differentially expressed genes.

In recent years, there has been renewed interest in natural products for the discovery and development of new anticancer drugs. Mushrooms have caught researchers’ attention due to their anticancer activity demonstrated in a variety of human cancer cells including breast, prostate and colorectal cancer cells *in vitro* (Hu et al., 2002; Stanley et al., 2005; Hsu et al., 2008). The anti-proliferative and apoptotic properties of mushrooms have been proposed relying on their tumor cell growth inhibitory activity (Sarangi et al., 2006; Israilides et al., 2008). White genius mushroom is a white strain of *H. marmoreus* (also known as bunashimeji and hon-shimeji) and is very popular edible mushroom in Taiwan. Originally, *H. marmoreus* was cultivated with a polypropylene plastic bottle in environmental control system in Japan. White genius mushroom was cultivated with a modified polypropylene plastic bags and environmental control system. *H. marmoreus* cultivated with the new methods was named “white genius mushroom” in Taiwan. The white genius mushroom was harvested when the stalk length is more than 15 cm and the yield of fruiting bodies of a cultivation bag is about 270–350 g. The mycelium incubation time of white genius mushroom is about 120 days, it needed about 26–30 days from primordial mushroom fruiting bodies formation to harvest. It has been reported that the extracts of *H. marmoreus* had an anticancer activity (Bao and You, 2011). However, the exact mechanism underlying *H. marmoreus*-produced anticancer effect remains to be elucidated. To identify the critical genes and pathways that are involved in the WGM extracts-induced cell death of Hep3B cells and to better understand the underlying mechanisms, the transcriptomes were analyzed in control and WGM extracts-treated cells using the next-generation sequencing technology.

Reactive oxygen species (ROS) are highly reactive species that are generated from oxygen metabolism. Excessive production of ROS may lead to oxidative stress, loss of cell function and ultimately cell death. ROS have been proved to be signaling molecules involved in the regulation of cell survival and cell death (Szatrowski and Nathan, 1991; Trachootham et al., 2009; You et al., 2014). It has also been reported that ROS play an important role in various signal transduction pathways of autophagy and apoptosis (Zhang et al., 2016; Dhanasekaran and Reddy, 2017). Particularly, producing ROS participated in the drug-induced cancer cell apoptosis and autophagy has been receiving increasing attention (Chen et al., 2008; Azad et al., 2009). Although autophagy has been considered as a cell survival mechanism, there is accumulating evidence for cross-talk in the regulation of apoptosis and induction of autophagy (Gump and Thorburn, 2011; Mukhopadhyay et al., 2014; Wei et al., 2014). Furthermore, the occurrence of autophagy can promote apoptosis and, thus, accelerate cell death (Rubinstein et al., 2011; Mukhopadhyay et al., 2014).

According to the report provided by the Ministry of Health and Welfare in Taiwan, liver cancer was the second leading cause of death in 2020. Since liver cancer presents a serious public health problem and economic burdens on the society, many investigators have exerted efforts to prevent the development of the cancer. Although *H. marmoreus* was found to have anticancer activities, there is no substantial evidence relating *H. marmoreus* to treatment of cancer. In this study, WGM extracts was examined for its anticancer and chemopreventive activities in hepatocarcinoma cells.

## Materials and Methods

### Materials

*N*-Acetylcysteine and glutathione were purchased from Sigma Chemical Company (St. Louis, MO, United States). 5-(and-6)-Chloromethyl-20,70-dichlorodihydrofluorescein diacetate (CM-H_2_DCFDA) was from Molecular Probes, Inc. (Eugene, OR, United States). 3-Methyladenine was from MedChemExpress (Monmouth Junction, NJ, United States). Antibodies to various proteins were obtained from the following sources: β-Actin, beclin 1, LC3B, p62 and p62p(S403) were purchased from GeneTex Inc. (Irvine, CA, United States). LC3B was purchased Sigma Chemical Company. Horseradish peroxidase-conjugated goat anti-mouse and -rabbit IgG were from Abcam.

### Preparation of White Genius Mushroom and Identification of the Chemical Compositions of White Genius Mushroom

White genius mushroom used in this study is from 8329 Farm and was harvested in September in Taiwan Changhua city. The botanical origin of white genius mushroom was identified by Dr. Wei-Sung Li (Plant Pathology Division, Taiwan Agricultural Research Institute, Council of Agriculture, Executive Yuan, Taiwan). White genius mushroom extracts were prepared as previously described (You et al., 2011). The yield of dry extract of white genius mushroom was about 5.6%. The chemical composition of the WGM extracts was analyzed using UPLC-MS/MS analyses, MS data processing and Molecular networking–GNPS (http://gnps.ucsd.edu). UPLC-MS/MS analysis and data procession were performed by the Metabolomics Core Facility, Agricultural Biotechnology Research Center, Academia Sinica, Taipei, Taiwan.

### Human Hepatocellular Carcinoma Cell Line Hep3B Cells

Human liver cancer cell line Hep3B was obtained from the Food Industry Research and Development Institute (Hsinchu, Taiwan) and routinely cultured as previously described (You et al., 2019).

### Total RNA Extraction

Total RNAs were isolated from control or WGM extracts-treated Hep3B cells with AllPure Total RNA Isolation Kit (AllBio Science Inc., Taiwan) according to the manufacturer’s descriptions. RNA concentration was quantified using a spectrophotometer at a wavelength of 260 nm.

### Gene Ontology and Kyoto Encyclopedia of Genes and Genomes Analysis of Differentially Expressed Genes

Gene ontology (GO) analysis was used to classify the gene function, and functions of a series of specific gene were calculated by hypergeometric distribution. The major biological functions of differentially expressed genes (DEGs) can be determined by GO enrichment analysis. The potential functions of the DEGs in biological process, molecular function and cellular component were predicted by GO analysis in this study. GO-TermFinder (v0.86, https://pubmed.ncbi.nlm.nih.gov/15297299/) was used to identify GO terms that annotate a list of DEGs with statistical significance adjusted by FDR (false discovery rate, adj. *p* < 0.05), and calculates the number of DEGs involved in each term. Pathway enrichment analysis performed in this study is based on KEGG (Kyoto Encyclopedia of Genes and Genomes) pathway units and used a hypergeometric test to identify the pathways of the DEGs that are significantly enriched against the transcriptome background. Pathway analysis was used to assess significant pathways that DEGs participated according to the KEGG database. In the KEGG pathway enrichment analysis, enriched pathways were identified according to FDR-adjusted *p*-value (adj. *p* < 0.05). Sequencing raw data was uploaded to NCBI Sequence Read Archive (SRA), and the accession ID is PRJNA813700.

### Mitochondrial Reductase Activity Assay (MTT Assay)

Mitochondrial reductase activity assay was performed as previously described (You et al., 2011). Cells were seeded at a density of 5 × 10^4^ cells per well onto a 12-well plate 48 h before being treated with drugs. The cells were incubated with 0.1% DMSO or with various indicated concentrations of WGM extracts for 24 h. After treatment, cells were incubated with 2.4 × 10^−4^ M 3-(4,5-dimethylthiazol-2-yl)-2,5-diphenyltetrazolium bromide (MTT) for 1 h at 37°C and then washed with PBS (phosphate-buffered saline). After solubilization in dimethylsulfoxide, absorbance was measured at 550 nm.

### Measurement of Reactive Oxygen Species

This study used CM-H_2_DCFDA to detect the intracellular generation of reactive oxygen species. Hep3B cells were incubated with vehicle alone or with various indicated concentrations of white genius mushroom extracts for 2, 4, and 6 h. After treatment, cells were loaded with 5 μM CM-H_2_DCFDA for 30 min and then washed with warm PBS. CM-DCF fluorescence was measured using a fluorometer (Thermo Scientific Fluoroskan Ascent FL; Helsinki, Finland) at an excitation wavelength of 485 nm and an emission wavelength of 538 nm.

### Protein Preparation and Western Blot Analysis

Protein preparation and Western blot analysis were performed as previously described (Lee et al., 2009). The proteins (50 µg) were separated by SDS-PAGE, and then electrotransferred onto Immobilon-P Transfer Membranes (Millipore, Bedford, MA, United States). Membranes were probed with protein-specific primary antibodies followed by secondary antibodies labeled with horseradish peroxidase. The primary antibodies used in this study were as follows. β-Actin, 1:5000; beclin 1, 1:3000; LC3B, 1:1000; p62, 1:3000; p62p(S403), 1:3000. β-Actin was used as an internal control. The secondary antibody was used at a dilution of 1:20,000 of HRP-conjugated goat anti-mouse IgG (for β-actin and p62) or horseradish peroxidase-conjugated goat anti-rabbit IgG (for beclin 1, LC3B and p62p(S403)).

### Data Analysis and Statistics

One-way ANOVA followed by Bonferroni post hoc test was used for analysis of difference between each experimental group. A *p* value <0.05 was considered significant.

## Results

### White Genius Mushroom Extracts Induced Cell Death of Hep3B Cells

**FIGURE 1:**
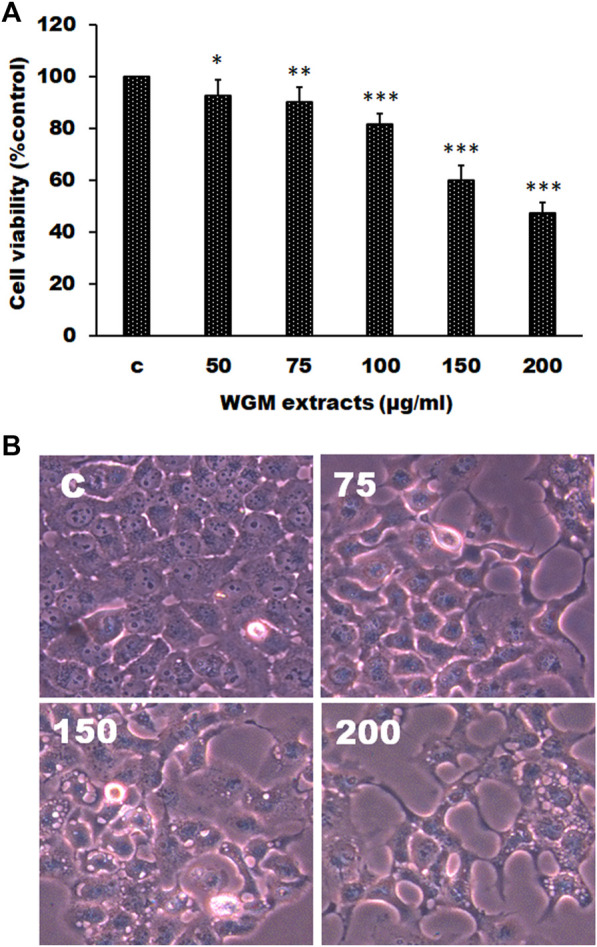
WGM extracts induced cell death of Hep3B cells. **(A)** Evaluation of cytotoxicity after incubation of Hep3B cells with WGM extracts. Hep3B cells were treated with 0.1% DMSO or with 50, 75, 100, 150 or 200 μg/ml of WGM extracts for 24 h. The cytotoxicity was evaluated by MTT assay. Data represent the mean percentage of control ±S.D. (*n* = 4). *p < 0.05, **p < 0.01, ***p < 0.001 vs. control group. **(B)** The effects of WGM extracts on cell morphology of Hep3B cells. Cells were incubated with 0, 75, 150 and 200 μg/ml WGM extracts for 24 h. After WGM extracts treatment, a phase-contrast image of the cells was fast taken (150X). (*n* = 4).

The effects of WGM extracts on cell death of human Hep3B liver cancer cells were determined by MTT assay. To evaluate the effect of WGM extracts on cell death of Hep3B cells, the cells were incubated with 50, 75, 100, 150, and 200 μg/ml of WGM extracts for 24 h. As shown in Figure 1A, cell viability was gradually reduced as the WGM extracts concentration increased. The IC_50_ (half maximal inhibitory concentration) of WGM extracts was about 175 μg/ml. In this study, the phenotypic characteristics of WGM extracts-treated Hep3B cells were also examined by microscopic inspection of overall morphology. It is noteworthy that treatment with 150 and 200 μg/ml WGM extracts for 24 h results in a significant increase in the formation of membrane-enclosed vacuoles within cells (Figure 1B). These results showed that WGM extracts had a significant cytotoxic effect on Hep3B cells. This study also treated Hep3B cells with 300 μg/ml of oyster mushroom, king oyster mushroom and black fungus extracts, which are also very popular edible mushroom in Taiwan. After treatment with oyster mushroom, king oyster mushroom and black fungus extracts for 24 h, the cells viability were 126.44 ± 7.98, 105.85 ± 6.28 and 94.14 ± 4.39%, respectively. The viable cells were measured by MTT assay and the fraction of viable cells were calculated by defining the absorption of cells without treatment of WGM extracts as 100%.

### Effects of White Genius Mushroom Extracts on mRNA Expression Profiles of Hep3B Cells

**FIGURE 2:**
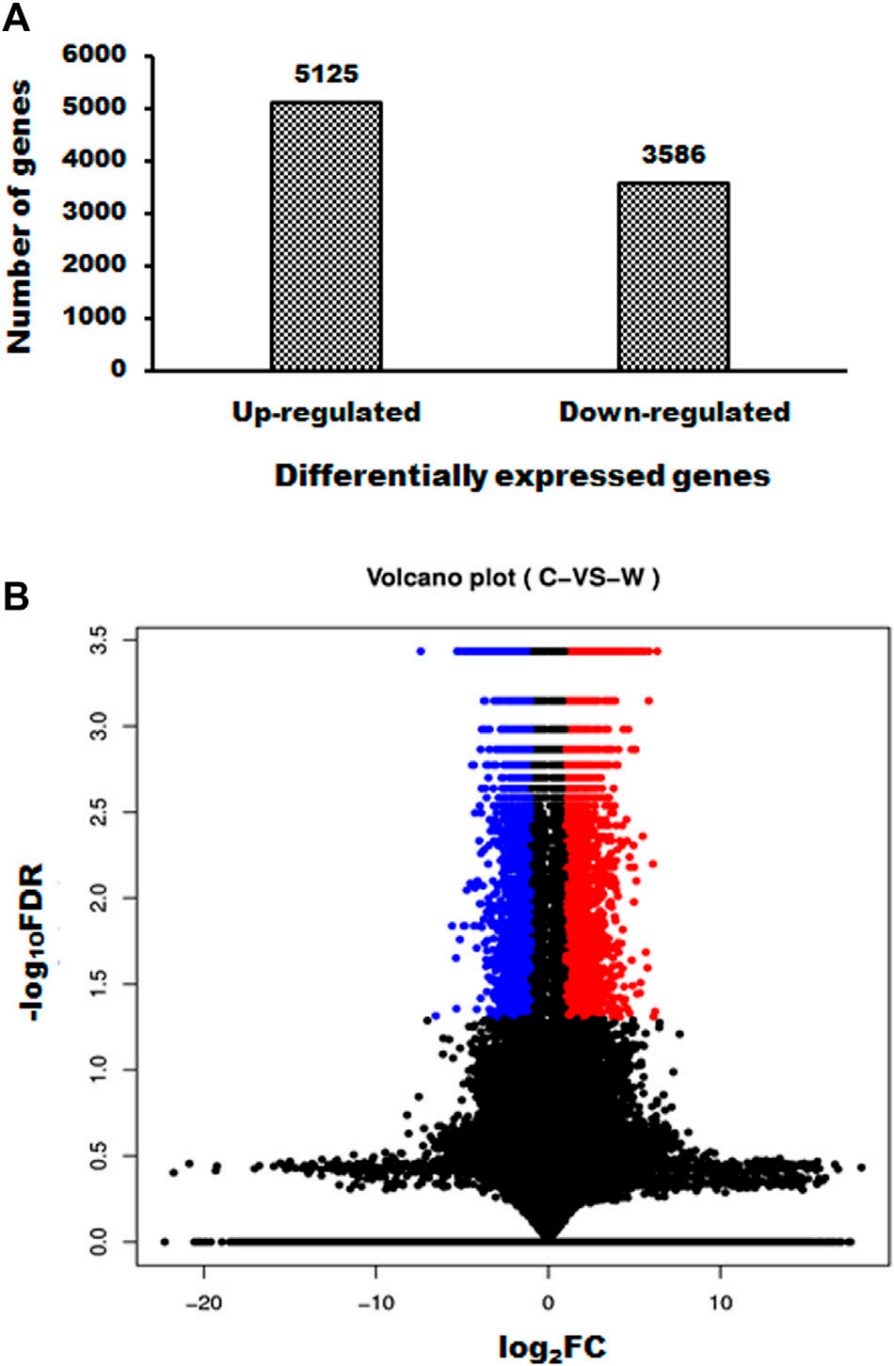
Summary of gene numbers that are significantly up or downregulated between WGM extracts-treated and control cells. **(A)** A total of 8711 genes (5125 upregulated and 3586 downregulated) was found to be differentially expressed between WGM extracts-treated and control samples. **(B)** Volcano Plot software was used to display the differentially expressed genes between WGM extracts-treated and control samples (*p* < 0.05, fold change >2). On the *x-axis*, the values of log_2_ of the fold change between the WGM extracts-treated and control samples are plotted. The y-axis shows the −log_10_ of the calculated probability (*p*-value). All results are representative of three independent experiments. (red, upregulated genes; blue, downregulated genes).

WGM extracts exhibits antitumor effects through an unknown mechanism. The purpose of the study was to investigate the mechanisms of WGM extracts-induced cell death of Hep3B cells. Next generation sequencing was used to analyze the gene expression profiles and identify the differentially expressed genes between control and WGM extracts-treated cells. Gene expression calculation was performed with Cuffdiff (v2.2.1) and HTSEQ (v0.6.1), and the results were further analyzed to determine genes with significant differential expression according to the criteria of fold change greater than 2 and FDR less than 0.05. Totally, 8711 differentially expressed genes were identified including 5125 upregulated genes and 3586 downregulated genes screened in 150 μg/ml WGM extracts-treated samples compared with control samples (Figure 2A). Volcano Plot software was used to display the DEGs between WGM extracts-treated and control samples (*p* < 0.05, fold change >2). The volcano plot can reflect the gene expression difference. Volcano plot was constructed by plotting the log_2_ of fold change of the WGM extracts-treated/control samples on the *x-axis*, whereas *y-axis* shows the negative log_10_ of the *p*-values where the data points with low *p*-values that are highly significant appear on the top of the plot (Figure 2B).

### Gene Ontology Term Enrichment Analysis and Kyoto Encyclopedia of Genes and Genomes Pathway Analysis of DEGs in White Genius Mushroom Extracts-Treated Hep3B Cells

**FIGURE 3:**
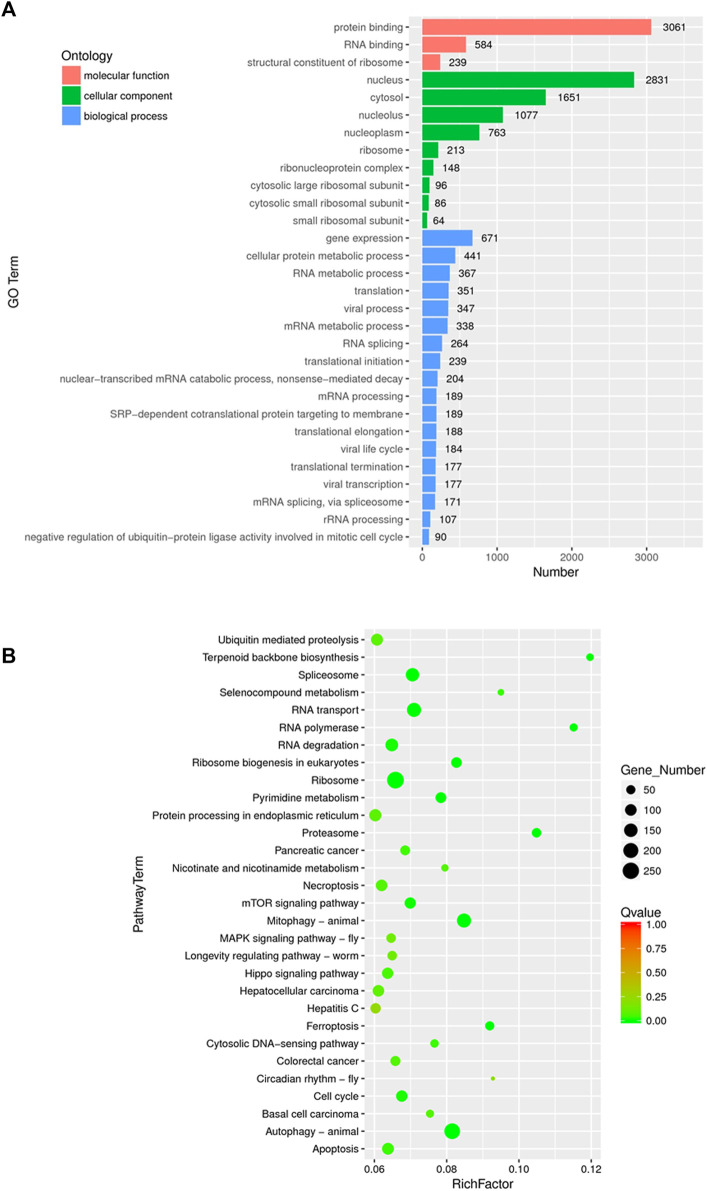
Functionally organized GO term and KEGG pathway enrichment of DEGs in WGM extracts-treated Hep3B cells. **(A)** Gene ontology (GO) analysis was used to predict the potential functions of the DEGs in biological process, molecular function and cellular component. Differentially expressed genes were selected based on more than 2-fold expression changes and the *p*-value was <0.05. **(B)** The bubble plot of KEGG pathway enrichments of the DEGs between WGM extracts-treated and control samples. Rich factors are presented as enrichment degree of DEGs. The *y-axis* shows the names of the enriched pathways. The Q-value, which is the name given to the adjusted *p*-values found using an optimized false discovery rate approach, is represented by color scale. Green represents high significance, while red represents low. The number of DEGs is indicated by the size of a circle. (*n* = 3)

GO annotation and KEGG pathway enrichment analysis were performed to systematically identify the functions and target signaling pathways of the differentially expressed genes (DEGs) in WGM extracts-treated cells. GO analysis showed that the DEGs were found to be involved in biological process, cellular component and molecular function (Figure 3A). Among the genes associated with molecular function, 3061 genes (2211 upregulated and 850 downregulated) were related to protein binding, 584 (481 upregulated and 103 downregulated) to RNA binding and 239 (230 upregulated and 9 downregulated) to structural constituent of ribosome (Figure 3A; Table 1). In the cellular component, genes encoding nucleus were predominant (2831 genes; 2050 upregulated and 781 downregulated), followed by cytosol (1651 genes; 1290 upregulated and 361 downregulated) and nucleolus (1077 genes; 835 upregulated and 242 downregulated) (Figure 3A; Table 1). Gene expression (671 genes; 597 upregulated and 74 downregulated), cellular protein metabolic process (441 genes; 374 upregulated and 67 downregulated) and RNA metabolic process (367 genes; 346 upregulated and 21 downregulated) were the dominant groups in terms of biological process (Figure 3A; Table 1). The enrichment of dysfunctional signaling pathways of the DEGs in WGM extracts-treated cells was screened by the KEGG pathway analysis, and the results revealed that 30 different pathways are involved in the WGM extracts-induced cell death (Figure 3B). A total of 18 KEGG pathways with enriched DEGs were shown in Table 2, among which the top seven enrichment pathways in a KEGG pathway analysis were the ribosome, autophagy, mitophagy, RNA transport, spliceosome, RNA degradation and apoptosis.

**TABLE 1:** Gene ontology analysis of differentially expressed genes in molecular function, cellular component and biological process.

GO term	Differentially expressed genes	
	Upregulated	Downregulated
Molecular function		
Protein binding	2211	850
RNA binding	481	103
Structural constituent of ribosome	230	9
Cellular component		
Nucleus	2050	781
Cytosol	1290	361
Nucleolus	835	242
Biological process		
Gene expression	597	74
Cellular protein metabolic process	374	67
RNA metabolic process	346	21

Hep3B cells were treated with vehicle alone or with 150 μg/ml WGM for 24 h. Gene ontology analysis of the differentially expressed genes between WGM-treated and control samples. (*n* = 3).

**TABLE 2:** KEGG pathway analysis of differentially expressed genes (DEGs).

Pathway ID	Genes with pathway annotation		*p*-value	Q-value
	DEGs (3735)	All genes (77737)		
ko03010	267 (7.15%)	4057 (5.22%)	9.06E-08	7.56E-06
ko04140	226 (6.05%)	2772 (3.57%)	4.77E-15	1.59E-12
ko03013	170 (4.55%)	2396 (3.08%)	2.16E-07	1.44E-05
ko04137	170 (4.55%)	2005 (2.58%)	4.48E-13	7.48E-11
ko03040	147 (3.94%)	2084 (2.68%)	1.82E-06	7.58E-05
ko03018	128 (3.43%)	1975 (2.54%)	3.07E-04	7.89E-03
ko04210	105 (2.81%)	1647 (2.12%)	1.60E-03	3.47E-02
ko04110	95 (2.54%)	1406 (1.81%)	4.18E-04	9.98E-03
ko04150	92 (2.46%)	1316 (1.69%)	1.64E-04	4.57E-03
ko00240	82 (2.20%)	1046 (1.35%)	7.20E-06	2.19E-04
ko03008	78 (2.09%)	943 (1.21%)	1.64E-06	7.58E-05
ko05212	62 (1.66%)	905 (1.16%)	2.39E-03	4.43E-02
ko03050	56 (1.50%)	534 (0.69%)	2.18E-08	2.43E-06
ko04216	50 (1.34%)	544 (0.70%)	5.42E-06	1.81E-04
ko04623	39 (1.04%)	509 (0.65%)	1.79E-03	3.52E-02
ko03020	35 (0.94%)	304 (0.39%)	7.09E-07	3.95E-05
ko00900	28 (0.75%)	234 (0.30%)	3.31E-06	1.23E-04
ko00450	19 (0.51%)	200 (0.26%)	1.66E-03	3.47E-02

Hep3B cells were treated with vehicle alone or with 150 μg/ml WGM for 24 h. KEGG pathway analysis of the differentially expressed genes between WGM-treated and control samples. ko03010, Ribosome; ko04140, Autophagy—animal; ko03013, RNA transport; ko04137, Mitophagy—animal; ko03040, Spliceosome; ko03018, RNA degradation; ko04210, Apoptosis; ko04110, Cell cycle; ko04150, mTOR signaling pathway; ko00240, Pyrimidine metabolism; ko03008, Ribosome biogenesis in eukaryotes; ko05212, Pancreatic cancer; ko03050, Proteasome; ko04216, Ferroptosis; ko04623, Cytosolic DNA-sensing pathway; ko03020, RNA polymerase; ko00900, Terpenoid backbone biosynthesis; ko00450, Selenocompound metabolism. (*n* = 3).

### White Genius Mushroom Extracts Induced Autophagy in Hep3B Cells

**FIGURE 4:**
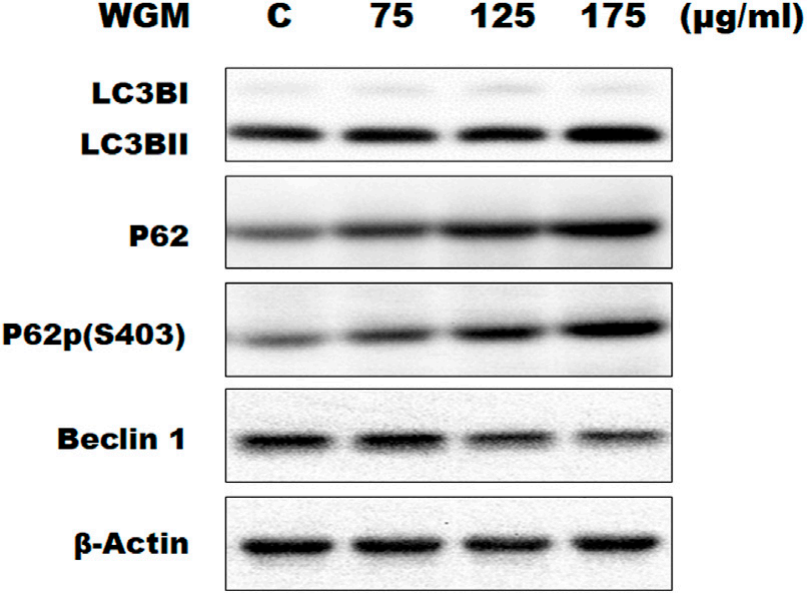
Effects of WGM extracts on the expression of autophagy-related genes in Hep3B cells. The effects of WGM extracts on the expression of autophagy-related genes were analyzed by Western blotting. Cells were treated with vehicle alone (C) or with 75, 125 or 175 μg/ml WGM extracts for 24 h. Protein samples were analyzed by SDS-PAGE (10% for beclin 1, p62 and p62p(S403), 12% for β-actin and 15% for LC3B), and then probed with primary antibodies followed by secondary antibodies. Results are representative of three independent experiments.

**FIGURE 5:**
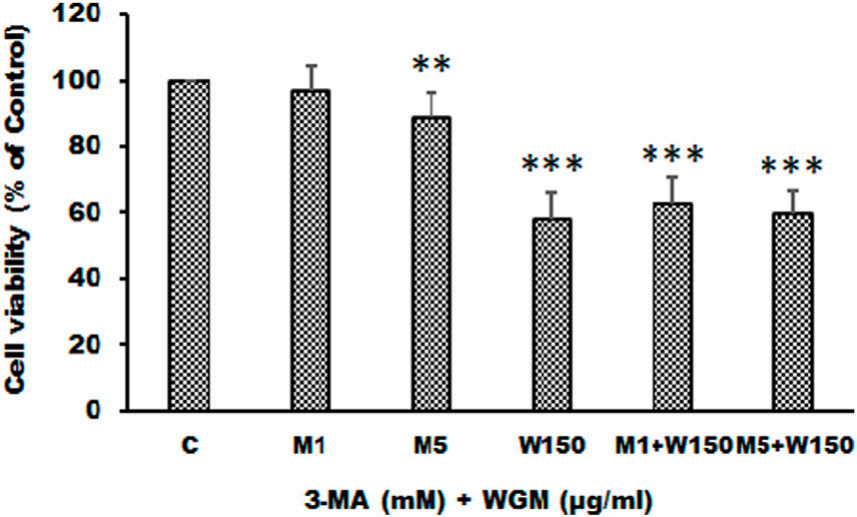
The effects of 3-MA on WGM extracts-induced cell death of Hep3B cells. Cells were pretreated with 1 or 5 mM 3-MA (M1 and M5) for 1 h and then treated with 0.1% DMSO (C) or 150 μg/ml WGM extracts (W150) for 24 h. The medium was not changed in the whole process of the experiment. After treatment, the viable cells were measured by MTT assay and the fraction of viable cells were calculated by defining the absorption of cells without treatment of WGM extracts as 100%. Data represent the mean percentage of control ±S.D. (*n* = 3). **p < 0.01, ***p < 0.001 compared to the control values.

KEGG pathway enrichment analysis was performed to systematically identify the functions of the DEGs in WGM extracts-treated cells. According to the KEGG pathway enrichment analysis, apoptosis (Q value = 3.47E-02), mitophagy (Q value = 7.48E-11) and autophagy (Q value = 1.59E-12) pathways were identified as significant in WGM extracts-treated cells compared to control cells (Table 2). Among the 3735 DEGs induced by WGM extracts, 105 DEGs are related to apoptosis, 170 DEGs are related to mitophagy and 226 DEGs are related to autophagy (Table 2). Based on the above data and reasons it suggested that WGM extracts should be able to induce autophagy in Hep3B cells in this study. Furthermore, WGM extracts-induced autophagy in Hep3B cells was supported by elevating the levels of autophagosome biogenesis-related proteins, such as LC3BII, p62 and p62p(S403) (Figure 4). Since autophagy-related gene beclin 1 plays a key role in autophagosome formation, the expression of beclin 1 in response to WGM extracts treatment was also examined. A dose-dependent reduction in beclin 1 protein expression was observed after 24 h treatment with WGM extracts (Figure 4). Interestingly, 1 or 5 mM of 3-methyladenine (3-MA), a well-known autophagic inhibitor, did not recover the cell death induced by 150 μg/ml WGM extracts (Figure 5). Furthermore, 3-MA (5 mM) alone slightly induced Hep3B cell death (Figure 5).

### White Genius Mushroom Extracts Induces Reactive Oxygen Species Generation in Hep3B Cells

**FIGURE 6:**
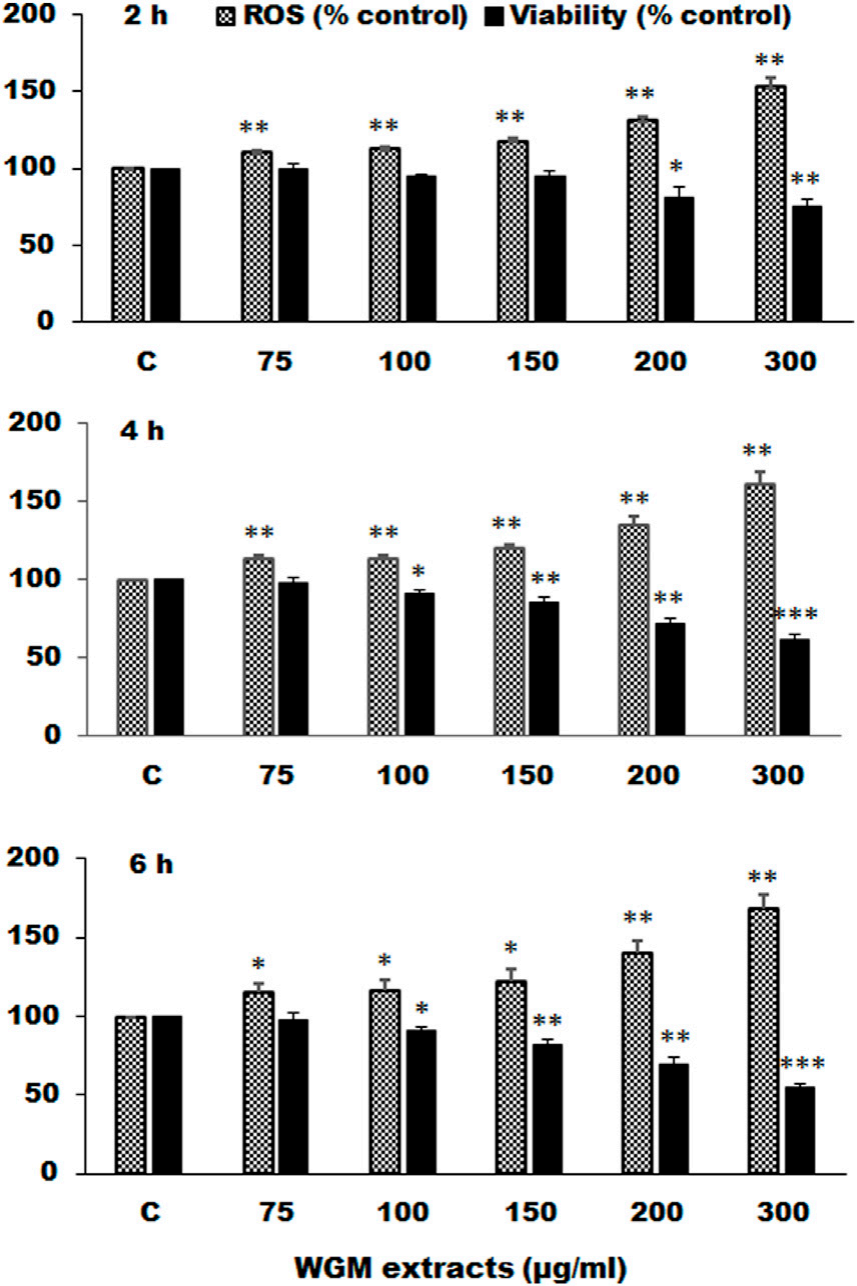
Effects of WGM extracts on reactive oxygen species (ROS) production in Hep3B cells. Hep3B cells were incubated with vehicle alone (C) or with various indicated concentrations of WGM extracts for 2, 4, and 6 h. After treatment, cells were loaded with 5 μM CM-H_2_DCFDA for 30 min. ROS fluorescence was measured using a multiwell plate reader at an excitation wavelength of 485 nm and an emission wavelength of 538 nm. MTT assay was also performed to determine the cell viability after treatment with WGM extracts for indicated times. Data represent the mean percentage of control ±S.D. (*n* = 4). *p < 0.05, **p < 0.01, ***p < 0.001 compared to the corresponding control values.

**FIGURE 7:**
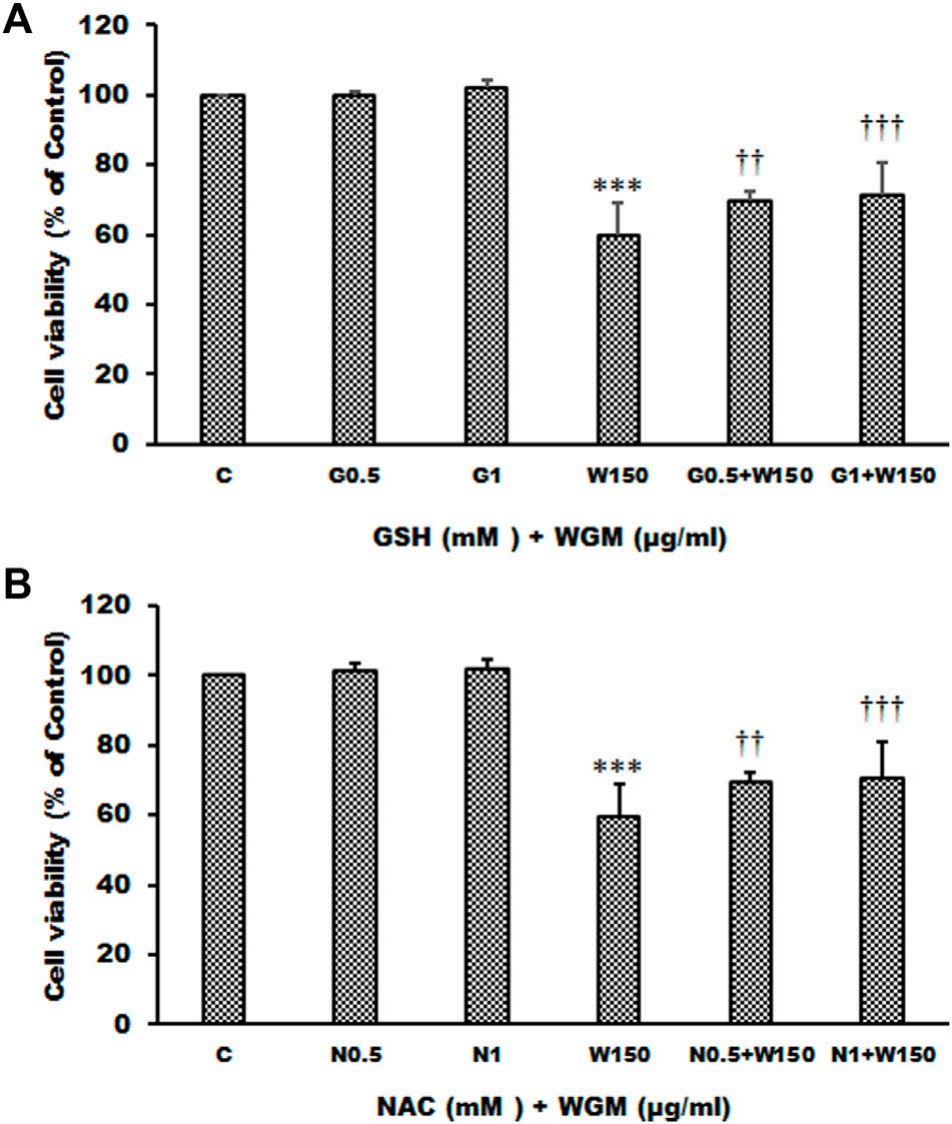
Effects of GSH or NAC on WGM extracts-induced cell death of Hep3B cells. **(A)** Cells were pretreated with 0.5 or 1 mM GSH (G0.5 and G1) for 1 h and then 150 μg/ml WGM extracts (W150) for 24 h. **(B)** Cells were pretreated with 0.5 or 1 mM NAC (N0.5 and N1) for 1 h and then 150 μg/ml WGM extracts (W150) for 24 h. The medium was not changed in the whole process of the experiment. After treatment, the viable cells were measured by MTT assay. Data represent the mean percentage of control ±S.D. (*n* = 4). ***p < 0.001 compared to the control values. ^††^ *p* < 0.01, ^†††^ *p* < 0.001 compared to the WGM extracts alone.

Many anti-cancer drugs have been shown to have an anti-tumor effects *via* ROS-dependent autophagic and apoptotic cell death. In order to demonstrate the role that ROS play in WGM extracts-induced cell death, intracellular ROS generation was examined by using an oxidant sensitive fluorescent probe, CM-H_2_DCFDA. As shown in Figure 6, the DCF fluorescence was measured in Hep3B cells treatment with 75, 100, 150, 200 or 300 μg/ml WGM extracts for 2, 4 and 6 h. There is a dose- and time-dependent increase in the production of ROS after treatment with WGM extracts in Hep3B cells (Figure 6). To further investigate whether the effect of WGM extracts on the change on Hep3B cell death could be linked to the production of ROS, reduced glutathione (GSH), a key antioxidant, and *N*-acetylcysteine (NAC), a scavenger of ROS and a precursor for the endogenous antioxidant glutathione, were used in this study. As shown in Figure 7, the present study found that GSH (0.5 or 1 mM) or NAC (0.5 or 1 mM) pretreatment significantly abolished the WGM extracts (150 μg/ml, 24 h)-induced cell death of Hep3B cells by MTT assay. Based on the above data, we suggested that there is a relationship between the production of ROS and cell death in Hep3B cells.

### Effects of GSH or NAC on White Genius Mushroom Extracts-Induced Membrane-Enclosed Vacuoles Formation in Hep3B Cells

**FIGURE 8:**
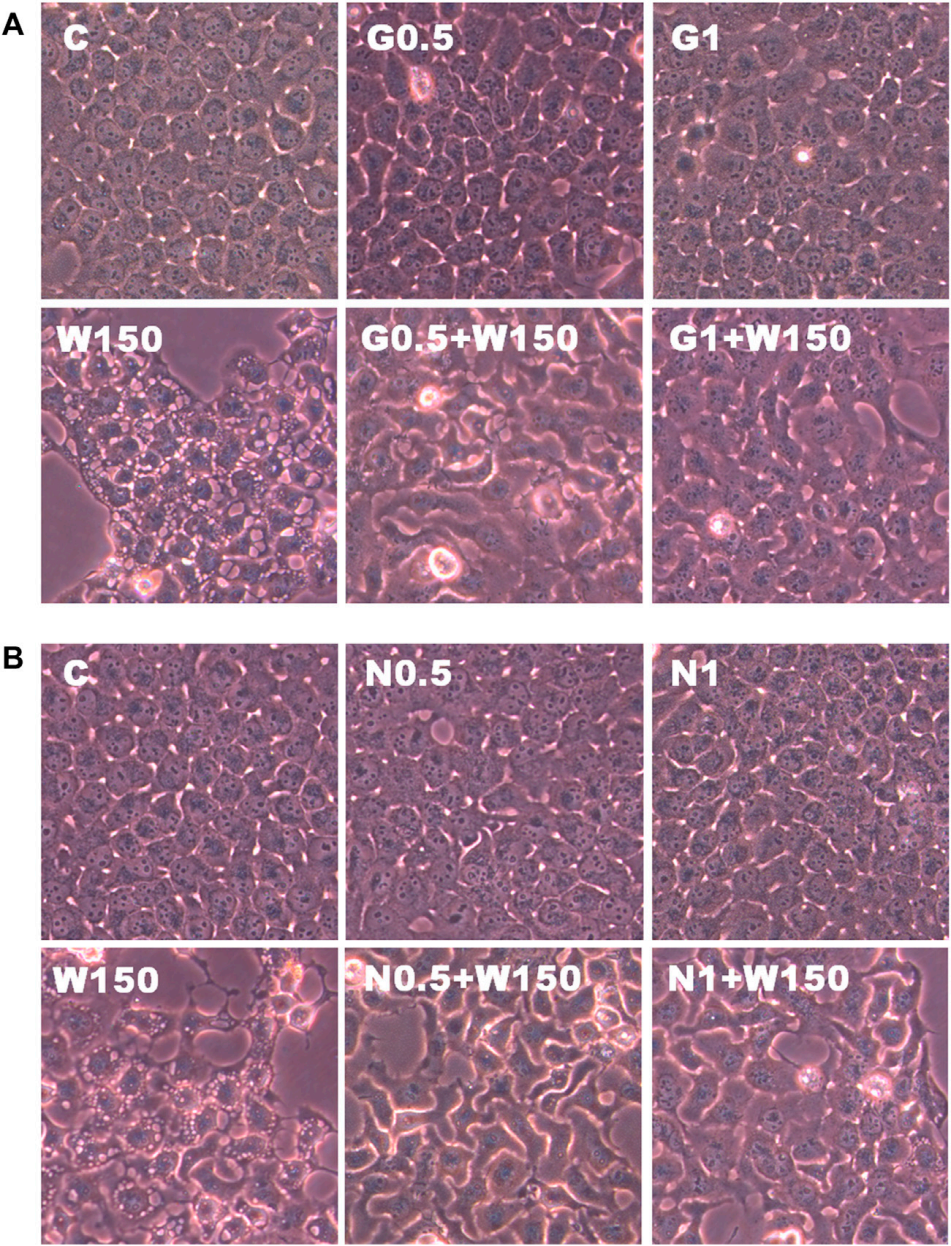
Effects of GSH or NAC on WGM extracts-induced changes in cell morphology of Hep3B cells. **(A)** Cells were pretreated with 0.5 or 1 mM GSH (G0.5 and G1) for 1 h and then 150 μg/ml WGM extracts (W150) for 24 h. **(B)** Cells were pretreated with 0.5 or 1 mM NAC (N0.5 and N1) for 1 h and then 150 μg/ml WGM extracts (W150) for 24 h. After treatment, the cells were fast taken a phase-contrast image (150X). Results are representative of three independent experiments.

**FIGURE 9:**
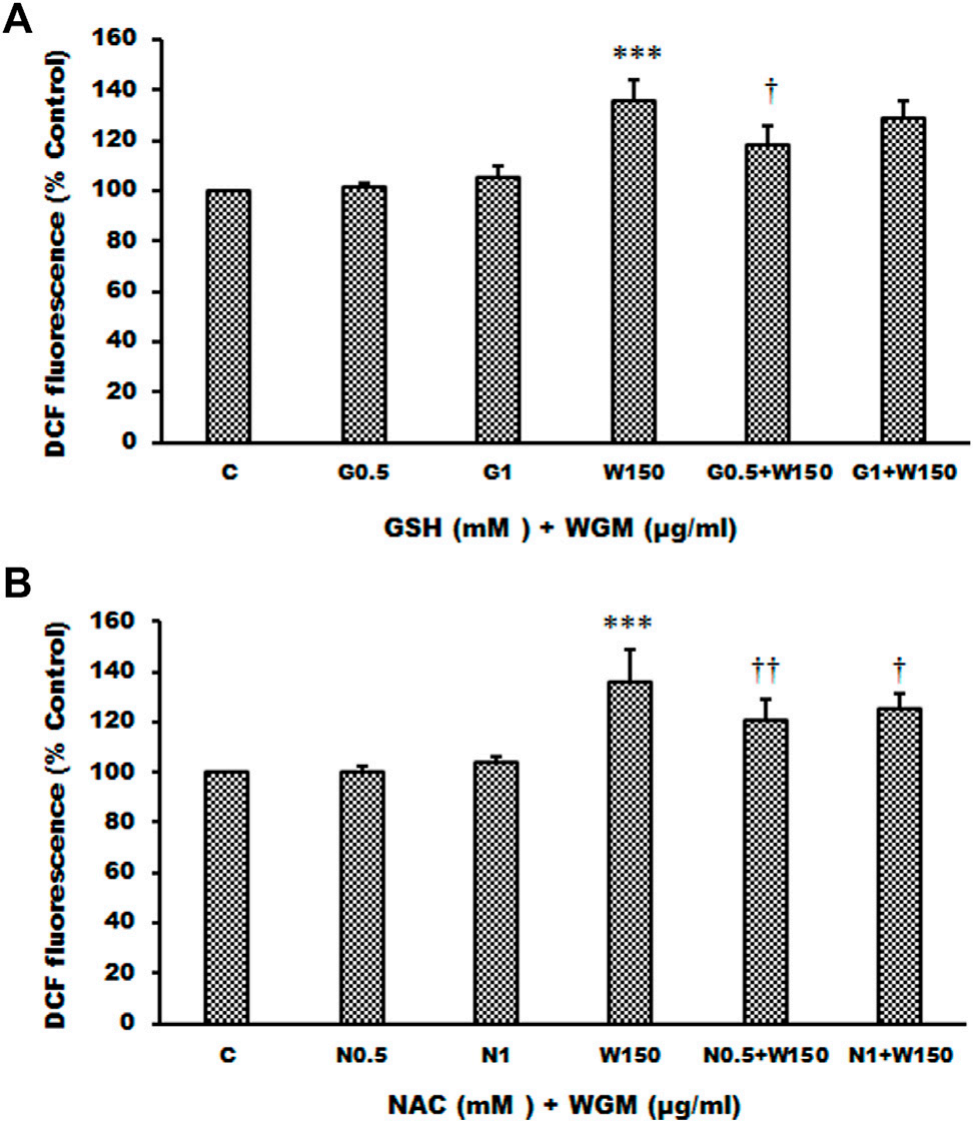
Effects of GSH or NAC on WGM extracts-induced ROS production of Hep3B cells. **(A)** Cells were pretreated with 0.5 or 1 mM GSH (G0.5 and G1) for 1 h and then 150 μg/ml WGM extracts (W150) for 2 h. **(B)** Cells were pretreated with 0.5 or 1 mM NAC (N0.5 and N1) for 1 h and then 150 μg/ml WGM extracts (W150) for 2 h. After treatment, cells were loaded with 5 μM CM-H_2_DCFDA for 30 min. ROS fluorescence was measured using a multiwell plate reader at an excitation wavelength of 485 nm and an emission wavelength of 538 nm. Data represent the mean percentage of control ±S.D. (*n* = 4). ***p < 0.001 compared to the control values. ^†^ *p* < 0.05, ^††^ *p* < 0.01 compared to the WGM extracts alone.

**FIGURE 10:**
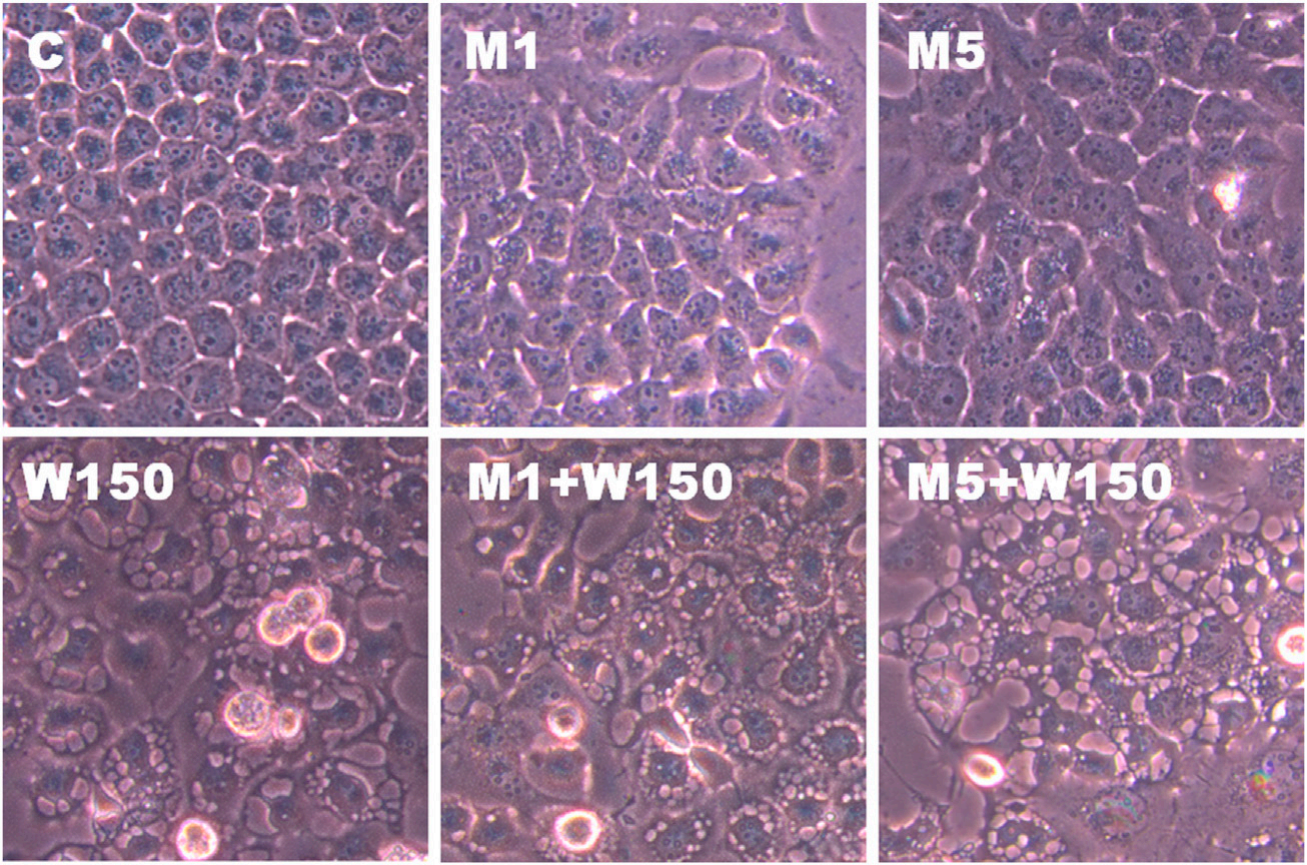
The effects of 3-MA on WGM extracts-induced membrane-enclosed vacuoles of Hep3B cells. Cells were pretreated with 1 or 5 mM 3-MA (M1 and M5) for 1 h and then treated with 0.1% DMSO (C) or 150 μg/ml WGM extracts (W150) for 24 h. After treatment, the cells were immediately photographed (150X). Results are representative of three independent experiments.

The present study further evaluated the relationship between the production of ROS and membrane-enclosed vacuoles formation induced by WGM extracts in Hep3B cells. The phenotypic characteristics of GSH and NAC on WGM extracts-induced membrane-enclosed vacuoles formation of Hep3B cells were also examined. Pretreatment GSH (0.5 or 1 mM) had significant effect on membrane-enclosed vacuoles formation induced by WGM extracts (150 μg/ml) in Hep3B cells (Figure 8A). As shown in Figure 8B, the pretreatment of Hep3B cells with 0.5 or 1 mM NAC for 24 h significantly abolished the WGM extracts (150 μg/ml, 24 h)-induced membrane-enclosed vacuoles of Hep3B cells, similar to GSH. To further examine whether GSH or NAC reduces vesicle formation through inhibition ROS production during WGM extracts-induced cell death, Hep3B cells were pretreated with NAC (0.5 and 1 mM) or GSH (0.5 and 1 mM) for 1 h and then 150 μg/ml of WGM extracts for 2 h. In this study, NAC had a more significant inhibitory effect than GSH on WGM extracts-mediated ROS production (Figure 9). Based on the above data, the preventive effect of NAC or GSH on WGM extracts-induced membrane-enclosed vacuoles formation seems to be relevant to the ROS production in Hep3B cells in this study. Furthermore, we guess that the membrane-enclosed vacuoles appearance of WGM extracts treated Hep3B cells is a phenomenon exhibited by autophagy. To further investigate whether the induction of vacuole by WGM extracts was a typical autophagic vacuole in Hep3B cells, the phenomena further confirmed by 3-MA, which is an autophagic inhibitor. The present study showed that 3-MA did not recover the membrane-enclosed vacuoles induced by WGM extracts (Figure 10). It seemed to suggest that there is no observable connection between the autophagy and vesicle formation.

### Identification of the Chemical Compositions of White Genius Mushroom Extracts

**FIGURE 11:**
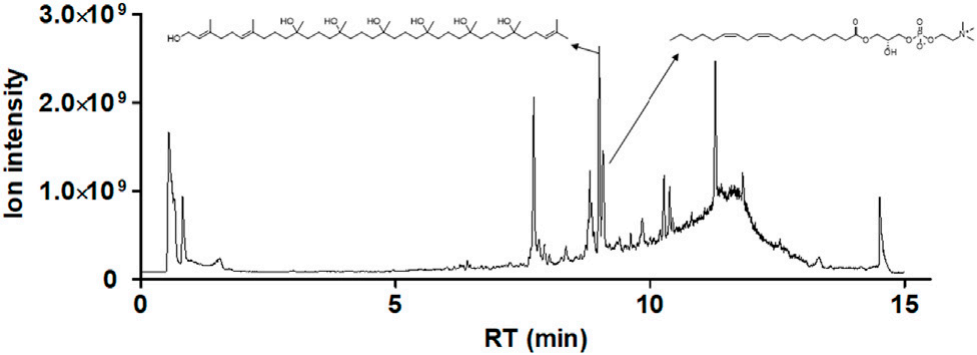
Identification of the chemical compositions of WGM extracts. The chemical compositions of the WGM extracts were analyzed using UPLC-MS/MS analyses, MS data processing and Molecular networking–GNPS (http://gnps.ucsd.edu). There are only two components of WGM extracts which were identified by UPLC-MS/MS analysis, MS data processing and Molecular networking–GNPS. The retention time of (2*E*,6*E*)-3,7,11,15,19,23,27,31,35-nonamethylhexatriaconta-2,6,34-triene-1,11,15,19,23,27,31-heptol and (18:2) lysophosphatidylcholine are 8.9 and 9.0 min, respectively.

In general, the crude extracts of natural products contain complex mixtures. Therefore, when the crude extract has anti-cancer activity, biologically active constituents with potential anticancer activity were fully expected to identify from the crude extracts. In this study, LC-MS/MS analysis, MS data processing and Molecular networking-GNPS were used to analyze the phytocompounds of WGM alcoholic extracts. Only (2*E*,6*E*)-3,7,11,15,19,23,27,31,35-nonamethylhexatriaconta-2,6,34-triene-1,11,15,19,23,27,31-heptol and (18:2) lysophosphatidylcholine (lysoPC), which are the larger node in the LC-MS/MS spectrum, were identified in the WGM alcoholic extracts. As shown in Figure 11, the retention time of (2*E*,6*E*)-3,7,11,15,19,23,27,31,35-nonamethylhexatriaconta-2,6,34-triene-1,11,15,19,23,27,31-heptol (*m/z* 739.64) and (18:2) lysophosphatidylcholine (*m/z* 520.34) are 8.9 and 9.0 min, respectively (https://gnps.ucsd.edu/ProteoSAFe/status.jsp?task=039cbc30e6c241de86368e21d24be32d, 5 October 2021).

## Discussion

White genius mushroom is a white strain of *H. marmoreus* (also known as bunashimeji and hon-shimeji) and is very popular edible mushroom in Taiwan. In this study, we explored the mechanisms of WGM extracts-induced cell death in Hep3B cancer cells. Recently, transcriptome profiling has been employed extensively to identify cellular states or to find genes with similar expression patterns (Wu et al., 2019; Shi et al., 2020). In general, GO and KEGG pathway enrichment analysis were subsequently conducted to elucidate cellular processes at the molecular level and further to investigate the key determinants for drugs-induced gene expression profiles. GO analysis is a commonly used method for the functional annotation of large amounts of genomic data (Gene Ontology Consortium, 2006). The KEGG pathways database is a comprehensive and well recognized database with a wide range of biochemical pathways (Kanehisa and Goto, 2000). The enrichment of dysfunctional signaling pathways was screened by the KEGG pathway analysis. In this study, next generation sequencing (NGS) technologies was used to examine differentially expressed genes between WGM extracts-treated and control cells. According to the result of NGS analysis, 8711 differentially expressed genes were identified including 5125 upregulated genes and 3586 downregulated genes screened in 150 μg/ml WGM extracts-treated samples compared with control samples. GO analysis showed that the DEGs were found to be involved in biological process, cellular component and molecular function. KEGG pathway enrichment analysis was performed in the present study to systematically determine the enrichment of signaling pathways of the differentially expressed genes in WGM extracts-treated cells. A total of 18 KEGG pathways with enriched DEGs were obtained. According to the KEGG pathway enrichment analysis, apoptosis, autophagy and mitophagy pathways were identified as significant in WGM extracts-treated cells compared to control cells. Among the 3675 DEGs induced by WGM extracts, 105 DEGs are related to apoptosis, 170 DEGs are related to mitophagy and 226 DEGs are related to autophagy. It suggested that WGM extracts might be able to induce autophagy in Hep3B cells in this study.

To further demonstrate the possible role of autophagy of WGM extracts-induced cell death, the present study examined the protein expression of the endogenous autophagy markers p62 and LC3B. P62 can shuttle ubiquitinated cargo for autophagic degradation that is used as a reporter of autophagy activity (Komatsu and Ichimura, 2010; Katsuragi et al., 2015). Phosphorylation of p62 on serine 403 (p62p(S403)) is thought to increase its binding affinity for ubiquitin chains and is a key component in the autophagic degradation of poly-ubiquitinated proteins (Katsuragi et al., 2015). LC3B has been widely used to be an autophagosomal marker for monitoring autophagy. LC3BII, the lipid modified form of LC3B, is believed to be involved in autophagosome membrane expansion and fusion events and be a standard marker for autophagosomes (Kabeya et al., 2000). In this study, WGM extracts induced a concentration-dependent accumulation of LC3BII, p62 and p62p(S403) proteins which are consistent with those of other studies reporting that an increase in LC3BII or p62 expression is required for autophagy induction (Puissant et al., 2010; Katsuragi et al., 2015; Martino et al., 2019). We then examined the protein levels of beclin 1, a key regulator of autophagosome formation (Kang et al., 2011). The protein levels of beclin 1 was gradually reduced as the WGM extracts concentration increased, indicating that WGM extracts-induced autophagy in Hep3B cell is a beclin 1-independent process. Although beclin 1 was often perceived to be a key player in autophagy execution and its knock down blocks autophagic cell death (Yue et al., 2003), accumulating evidences showed that beclin 1-independent autophagy is invariably associated with cell death (Scarlatti et al., 2008; Shrivastava et al., 2011).

Excessive production of ROS may lead to oxidative stress, loss of cell function and ultimately cell death. In fact, many anti-cancer drugs have been shown to have an anti-tumor effects *via* ROS-dependent autophagic and apoptotic cell death in various cancer cells (Chen et al., 2008; Azad et al., 2009). It has also been reported that reactive oxygen species play an important role in various signal transduction pathways, such as JNK-signaling and NF-κB pathway, of autophagy and apoptosis (Zhang et al., 2016; Dhanasekaran and Reddy, 2017). Autophagy and particularly mitophagy have been shown to play an essential role in reducing the mitochondrial ROS production, thereby allowing mitochondria to avoid oxidative damage (Kurihara et al., 2012; Bin-Umer et al., 2014). In this study, we demonstrated that autophagy, mitophagy and apoptosis pathways were involved in WGM extracts-induced Hep3B cell death according to the KEGG pathway enrichment analysis. Although beclin 1 expression was required for ROS-induced autophagy (Azad et al., 2009), it has also been reported that anticancer agents can induce ROS-mediated beclin 1-independent autophagy in various cancer cell types (Al Dhaheri et al., 2014; Martino et al., 2019). The reduced levels of beclin 1 are believed to inhibit autophagy and prevent the turnover of damaged mitochondria, leading to further production of ROS and oxidative stress (Kurihara et al., 2012; Bin-Umer et al., 2014). Since mitophagy and a dose-dependent reduction in beclin 1 protein expression were observed after 24 h treatment with WGM extracts, the ROS production was determined in this study. The present study demonstrated that there was a dose- and time-dependent increase in the production of ROS after treatment with WGM extracts in Hep3B cells. It seemed to suggest that mitophagy cannot effectively eliminate the mitochondrial damage during WGM extracts-induced cell death, which leads to further production of ROS and oxidative stress, resulting in a dramatic loss in cell viability.

To further investigate whether the effect of WGM extracts on the cell death of Hep3B cells could be linked to the production of ROS, reduced glutathione, a key antioxidant, and *N*-acetylcysteine, a precursor of glutathione, were used in this study. The present study demonstrated that glutathione or *N*-acetylcysteine significantly reversed the WGM extracts-mediated Hep3B cell death. It suggested that ROS production be involved in WGM extracts-induced Hep3B cancer cell death. The phenotypic characteristics of WGM extracts-treated Hep3B cells were also examined by microscopic inspection of overall morphology. It is noteworthy that treatment with WGM extracts results in a significant dose-dependent increase in the formation of membrane-enclosed vacuoles within cells. We have observed that WGM extracts at 150 μg/ml causes the accumulation of big cytoplasmic vacuoles and cell death in Hep3B cells, while pretreatment with glutathione or *N*-acetylcysteine reduced vacuole formation and cell death in the cells. Furthermore, a reduction in WGM extracts-mediated ROS production was observed in the experiments of glutathione or *N*-acetylcysteine pretreatment. Based on the above data, we guess that vacuole formation and cell death are related to the production of ROS during the WGM extracts-induced cell death.

We further examined whether WGM extracts-induced the formation of membrane-enclosed vacuoles is associated with the induction of autophagy by WGM extracts in Hep3B cells. In this study, inhibitor of autophagy, 3-methyladenine (3-MA), was used to confirm whether membrane-enclosed vacuoles induced by WGM extracts were related to autophagy. 3-MA has been widely used as autophagic inhibitor due to its inhibitory effect on autophagosome formation. In this study, 3-MA cannot relieve the membrane-closed vacuoles induced by WGM extracts. It seems to indicate that there is no observable connection between the membrane-enclosed vacuoles induction and autophagy. Our results also showed that 3-MA had no effect on the WGM extracts-induced cell death, indicating autophagy is probably not the primary signal involved in WGM extracts-induced cell death. Furthermore, 3-MA alone slightly induced Hep3B cell death. Although autophagy has been considered as a cell survival mechanism, there is accumulating evidence for cross-talk in the regulation of apoptosis and induction of autophagy (Gump and Thorburn, 2011; Mukhopadhyay et al., 2014; Wei et al., 2014). Furthermore, the occurrence of autophagy can promote apoptosis and, thus, accelerate cell death (Rubinstein et al., 2011; Mukhopadhyay et al., 2014). Although autophagy has been considered as cell survival mechanism, our study had shown that autophagy involved in WGM extracts-induced Hep3B cell death. Since 3-MA cannot alleviate or inhibit cell death caused by WGM extracts, we guessed that autophagy induced by WGM extracts could be an indication that the cells attempted to survive through induction autophagy in this study.

LysoPC is a monoglycerophospholipid in which a phosphorylcholine moiety is attached to the *sn*-3 position of glycerol. LysoPC can have different long-chain fatty acids, which is the 16, 18 and 20 carbon atoms, bonded to the *sn*-1 position of glycerol. (18:2(9Z,12Z)) lysoPC is a linoleic acid at the *sn*-1 position. LysoPC is normally generated from phosphatidylcholine (PC) through the catalysis of phospholipase A2 (PLA2) and is present in cell membranes, oxidized lipoproteins and atherosclerotic tissues. Previous studies had demonstrated that the downregulation of PC and upregulation of lysoPC could induce cell apoptosis (Anthony et al., 1999; Cui and Houweling, 2002; Takahashi et al., 2002). In the research of the antitumor effects of PP242, an inhibitor of mechanistic target of rapamycin (mTOR), in a colon cancer xenograft mouse model, Rashid et al. (2020) indicated that lysoPC (18:2) in the PP242-treated mice was significantly increased after mTOR inhibition. It has also been suggested that lysoPC (16:0) induces apoptosis in human endothelial cells through a p38-MAPK-dependent pathway (Takahashi et al., 2002). (2*E*,6*E*)-3,7,11,15,19,23,27,31,35-nonamethylhexatriaconta-2,6,34-triene-1,11,15,19,23,27,31-heptol is a polyhydroxy isoprenoid and has been described to be a novel structural class of inhibitors of the human neurokinin-1 (NK-1) and neurokinin-2 (NK-2) receptors (Hegde et al., 1997). The neurokinin receptors, also known as tachykinin receptors, have played an important role in the regulation of a variety of fundamental biological processes such as neuronal activity, cell proliferation, inflammation, etc (Campos and Calixto, 2000; Pennefather et al., 2004; Muñoz et al., 2019). Recently, it has been reported that the NK-1 receptor (NK1R) played a pivotal in the development of cancer, including pancreatic cancer, thyroid cancer, gastrointestinal cancer and breast carcinoma (Muñoz and Coveñas, 2014; Muñoz and Coveñas, 2016; Ebrahimi et al., 2020; Isorna et al., 2020). Furthermore, new evidence suggested that NK1R is overexpressed in human hepatoma and can be antagonized to exhibit a significant inhibitory effect on the proliferation of cancer cells *in vivo* and *in vitro* (Garnier et al., 2016). Therefore, NK-1R is currently considered to be an important target for the treatment of hepatoblastoma. Based on the above data and reasons, in this study, we have tested the hypothesis that these two anti-cancer components of WGM extracts may be the reason of cell death caused by WGM extracts.

## Conclusion

White genius mushroom is very popular edible mushroom in Taiwan. We hope that WGM may be developed into a dietary supplement or dietary chemopreventive agent for the cancer treatment. For defining WGM as a promising chemopreventive agent against human liver cancer, we explored the mechanisms of WGM extracts-induced cell death in human hepatoma Hep3B cancer cells. Next generation sequencing was used for identifying the target pathways associated with WGM extracts in Hep3B cells. According to the KEGG pathway enrichment analysis, autophagy, mitophagy and apoptosis pathways were identified as significant in WGM extracts-treated cells compared to control cells. In this study, protein expression of the endogenous autophagy markers LC3BII, p62 and p62p(S403) was elevated subsequent to WGM extracts treatment, suggesting that WGM extracts indeed induce autophagy in Hep3B cells. The present study also demonstrated that WGM extracts-induced cell death and ROS production is partially inhibited by GSH or NAC pretreatment in Hep3B cells, but membrane-enclosed vacuoles formation is completely prevented. Therefore, there is observable connection between the production of ROS and membrane-enclosed vacuoles formation or cell death. In the current study, we provided evidence that WGM extracts induced beclin 1-independent autophagic cell death through ROS generation.

## Data Availability

The datasets presented in this study can be found in online repositories. The names of the repository/repositories and accession number(s) can be found below: National Center for Biotechnology Information (NCBI) BioProject database under accession number PRJNA813700.

## Abbreviations

CM-H2DCFDA, 5-(and-6)-chloromethyl-20,70-dichlorodihydrofluorescein diacetate; DEGs, differentially expressed genes; FDR, false discovery rate; GO, gene ontology; GSH, reduced glutathione; KEGG, kyoto encyclopedia of genes and genomes; 3-MA, 3-methyladenine; MTT, 3-(4,5-dimethylthiazol-2-yl)-2,5-diphenyltetrazolium bromide; NAC, N-acetylcysteine; NGS, next generation sequencing; ROS, reactive oxygen species; WGM, white genius mushroom.
